# Mechanism of circRNA_4083 Circularization and Its Role in Regulating Cell Viability

**DOI:** 10.3390/ani15111527

**Published:** 2025-05-23

**Authors:** Wenhao Li, Ting Yang, Haojie Wang, Hao Bai, Guobin Chang, Lingling Qiu

**Affiliations:** 1 College of Animal Science and Technology, Yangzhou University, Yangzhou 225009, China; l2823667541@163.com (W.L.); yt1144736744@163.com (T.Y.); hjwang2024@163.com (H.W.); 2 Joint International Research Laboratory of Agriculture and Agri-Product Safety, The Ministry of Education of China, Institutes of Agricultural Science and Technology Development, Yangzhou University, Yangzhou 225009, China; bhowen1027@yzu.edu.cn

**Keywords:** circRNA_4083, circular RNA biogenesis, cell viability, chicken

## Abstract

Circular RNAs (circRNAs), a class of covalently closed non-coding RNAs, are pivotal regulators of gene expression and contributors to disease pathogenesis. This study elucidated the biogenesis, functional significance, and regulatory network of circRNA_4083, a novel exon-derived circRNA originating from exons 22 and 23 of the *MSH3* gene in chicken. Through comprehensive molecular characterization—including Sanger sequencing, RNase R digestion assays, and subcellular localization—we confirmed the robust stability and predominant cytoplasmic localization of circRNA_4083 across diverse chicken tissues. Mechanistic investigations revealed that reverse complementary sequences within flanking intronic regions are indispensable for its circularization, as demonstrated by overexpression plasmids (#1–#4) in DF-1 cells. Functional analyses demonstrated that circRNA_4083 significantly inhibited cell apoptosis and increased cellular viability. Integrative bioinformatics approaches predicted a competing endogenous RNA (ceRNA) network comprising 12 miRNAs and 2132 target genes (FDR < 0.05), with significant enrichment in pathways critical to genomic stability, including non-homologous end joining (NHEJ) and ubiquitin-mediated proteolysis. These findings position circRNA_4083 as a key modulator of cellular viability and genomic integrity, with potential implications for avian leukosis virus-J (ALV-J) pathogenesis and resistance breeding strategies. This work advances our understanding of circRNA-driven regulatory mechanisms in avian species and underscores their relevance in poultry health.

## Simple Summary

This study investigated circRNA_4083, which is a stable circular RNA which originates from the *MSH3* gene of chicken. It is highly expressed in cardiac, pulmonary and renal tissues. Its biogenesis depends on flanking genomic sequences. CircRNA_4083 overexpression reduced apoptosis and enhanced cell proliferation, indicating its role in promoting cellular viability. Bioinformatics analysis indicates that circRNA_4083 interacts with RNA-binding molecules and regulates pathways that are crucial for genomic stability, possibly protecting the genome from damage. These findings underscore its importance in maintaining cellular health. By elucidating circRNA-driven mechanisms, this work advances the understanding of non-coding RNA functions in poultry biology, offering insights for developing strategies to improve disease resilience and productivity in agricultural practices.

## 1. Introduction

Circular RNAs (circRNAs) are a unique class of non-coding RNA molecules characterized by covalently closed loop structures, predominantly localizing in the cytoplasm or exosomes, with a minor fraction residing in the nucleus. These molecules exhibit high stability and tissue-specific expression patterns. In eukaryotic cells, circRNAs function as miRNA sponges to regulate gene expression, participate in cellular metabolism and protein translation, and modulate cell cycle progression and stress responses [1,2]. Notably, circRNAs serve as promising biomarkers for various diseases, including cancers, diabetes, and other pathological conditions, and may contribute to disease pathogenesis [3]. Piwecka et al. demonstrated that deletion of circRNA loci disrupted miRNA homeostasis, impairing normal brain function [4]. Chen et al. revealed that circRNAs could recruit translation initiation factors through specific sequence elements to initiate protein synthesis [5]. Yang et al. identified that circFBXW7 encoded a novel protein in human glioma cell lines U251 and U373 [6]. In hepatocellular carcinoma, circRNA_MTO1 antagonizes the oncogenic miR-9 by blocking its regulation of downstream targets, thereby suppressing tumor progression [7]. Wang et al. reported that circLMTK2 acts as a sponge for miR-150-5p to promote gastric cancer proliferation and metastasis [8]. Liu et al. proposed elevated levels of hsa_circRNA_101015, hsa_circRNA_101211, and hsa_circRNA_103470 in patient serum as novel diagnostic biomarkers for acute pancreatitis [9].

In the previous study, we performed whole transcriptome sequencing on the spleen tissues of Silkie chickens infected with J-type avian leukosis virus (ALV-J) and identified differentially expressed circular RNA_4083 [10]. We hypothesized that this circRNA might play a significant role in ALV-J viral infection. circRNA_4083 was formed by the cyclization of exons 22 and 23 of the *MSH3* gene. The MutS Homolog 3 (*MSH3*) gene is a member of the DNA mismatch repair (MMR) gene family. The MSH3 protein encoded by this gene specifically repairs large insertion–deletion errors and microsatellite sequences during DNA replication, thereby maintaining genomic stability [11,12,13]. The *MSH3* gene has been implicated in multiple biological processes, including cancer development, neurodegenerative diseases, and genomic stability [14,15,16,17,18]. For example, O’Reilly D et al. demonstrated that inhibiting *MSH3* could reduce CAG repeat instability and pathological changes in Huntington’s disease mouse models [19]. Keogh N et al. investigated the roles of MutSβ and *MSH3* in CTG•CAG repeat expansions associated with various neurodegenerative diseases [20].

This study aims to investigate the presence of circRNA_4083 and predict its potential regulatory mechanisms, thereby establishing a foundation for exploring the role of circRNAs in the infection process of ALV-J.

## 2. Materials and Methods

### 2.1. Cell Isolation and Culture

Isolation and culture of chicken embryo fibroblast (CEF) cells: CEF cells were isolated from 10-day-old chicken embryos. Muscle tissues were dissected from the skin and skeleton, followed by digestion with 0.25% trypsin-EDTA (Gibco, code no. 25200072, Grand Island, NY, USA) at 37 °C for 20 min. The digestion was terminated by adding complete growth medium, and the suspension was centrifuged at 1000 rpm for 5 min. Cells were resuspended in 1 mL DMEM, filtered through a 70 μm cell strainer, counted, and seeded into 25 cm^2^ culture flasks. Cells were maintained in a temperature incubator (37 °C with 5% CO_2_).

DF-1 cell culture: Douglas Foster-1 (DF-1) cells (acquired from the American Type Culture Collection, code no. CRL-3586, Manassas, VA, USA) were cultured in DMEM medium (Cytiv, code no. SH30243.01, Marlborough, MA, USA) supplemented with 10% fetal bovine serum (Gibco, code no. 10099141C) and 1% penicillin–streptomycin antibiotic (Gibco, code no. 15140122), and maintained at 37 °C, 5% CO_2_, and 60–70% relative humidity. (The DF-1 cell line was tested for mycoplasma before use, and the result was negative).

### 2.2. Total RNA Extraction and Quality Assessment

Tissues, including the heart, liver, spleen, lung, kidney, lymph, and bursa, were collected from three adult roosters of the Silkie fowl breed (supplied by Lihua Animal Husbandry Co. Ltd, Changzhou, China.) and stored at −80 °C for subsequent total RNA extraction. Total RNA was extracted using TRIzol reagent (Thermo Fisher Scientific, code no. 15596026CN, Waltham, MA, USA). RNA purity and concentration were measured using a NanoDrop ND-1000 spectrophotometer (Thermo Fisher Scientific). RNA integrity was confirmed by A260/280 ratios (1.8–2.1) and A260/230 ratios (>2.0).

### 2.3. Validation of circRNA_4083

Convergent and divergent primers were designed based on the NCBI reference sequence of *MSH3* (Accession: NC_052572.1) and circRNA_4083. PCR amplification was performed using genomic DNA (gDNA) and cDNA from CEF cells to confirm circRNA_4083 existence and splicing junctions. All PCR products were sequenced by Tsingke Biotech (Beijing, China).

### 2.4. RNase Digestion

For RNase R treatment, 2.5 μg of total RNA was incubated with 3 U/μg of RNase R (GENESEED, code no. R0301, Guangzhou, China) or water without RNase (control) at 37 °C for 30 min, and then heated at 70 °C for 10 min to inactivate RNase R. The total RNA before and after RNase R treatment was reverse transcribed into cDNA using Hi Script IV RT Super Mix for qPCR (Vazyme, code no. R433-01, Nanjing, China). Then, RT-qPCR was performed using the cDNA to detect the expression levels of circRNA_4083 and its source gene *MSH3*.

### 2.5. qRT-PCR Analysis

cDNA was synthesized using Hi Script^®^ IV RT Super Mix for qPCR (Vazyme, code no. R423-01) with gDNA wiper. qRT-PCR was conducted with a Taq Pro Universal SYBR^®^ qPCR Master Mix (Vazyme, code no. Q712-02) on a QuantStudio™ 5 Real-Time PCR System (Thermo Fisher Scientific). Primers were designed using Primer 5.0 (PREMIER Biosoft, San Francisco, CA, USA) and Oligo 7 (Molecular Biology Insights, San Francisco, CA, USA) with GAPDH as the endogenous control. Reactions were performed in triplicate (primer sequences listed in Table 1).

### 2.6. Subcellular Localization

Nuclear and cytoplasmic fractions were extracted using the NE-PER nuclear and cytoplasmic extraction reagents (Thermo Fisher Scientific, code no. 78835). Total RNA from whole-cell lysates, tissues, or the nuclear and cytoplasmic fractions was isolated using TRIzol reagent. The extracted RNA was then subjected to quantitative PCR (qPCR) to detect the expression of circRNA_4083 in the nucleus and cytoplasm, respectively.

### 2.7. Plasmid Construction

The overexpression vector pcDNA3.1-circRNA_4083 was constructed via recombinant cloning. The circRNA-4083 sequence, the upstream and downstream sequences of exons 22 and 23, were amplified with PrimeSTAR^®^ Max DNA Polymerase (TaKaRa, code no. R047S, Kyoto, Japan) (primer sequences listed in Table 2). The linearized vector was digested with KpnI (New England Biolabs, code no. R3142V, Ipswich, MA, USA) and XhoI (New England Biolabs, code no. R0146V) in a 50 μL reaction (1 μg plasmid, 1 μL each enzyme, 10× buffer, 37 °C for 15 min). PCR products were confirmed by electrophoresis and purified using the TaKaRa Mini BEST Agarose Gel DNA Extraction Kit (TaKaRa, code no. 9762) Finally, the target fragment was combined with the linearized vector (pcDNA3.1) using the Clon Express^®^ Ultra One Step Cloning Kit (Vazyme, code no. C115-01) for homologous recombination.

### 2.8. Cell Transfection

The siRNAs targeting circRNA_4083 (s1-circRNA_4083, s2-circRNA_4083) and an siRNA negative control (si-NC) were designed and synthesized by Gene Pharma (Shanghai, China). The corresponding siRNA sequences are provided in Table 3. For transfection, cells were plated into 12- or 96-well plates and cultured to 70–80% confluency. siRNA transfection was performed using Lipofectamine™ RNAiMAX (Thermo Fisher Scientific, code no. 13778150), while plasmid transfection was performed using Lipofectamine™ 2000 (Thermo Fisher Scientific, code no. 11668019), both in strict accordance with the manufacturer’s protocols. All experiments included triplicate wells to ensure reproducibility. The empty vector-transfected control group was included to control for non-specific effects associated with the vector backbone. Following transfection, cells were maintained under standard culture conditions for 24–48 h prior to functional analyses.

### 2.9. Cell Viability Assay

Cell proliferation was assessed using a CCK-8 Kit (Vazyme, code no. A311-01). Cells were seeded in 96-well plates, pre-incubated for 8 h, and treated. Three groups were included: a blank (medium only), a control (untreated cells), and an experimental group. After treatment (24–72 h), 10 μL CCK-8 reagent was added per well, incubated for 1–4 h, and absorbance at 450 nm was measured. Cell viability (%) = [(Experimental − Blank)/(Control − Blank)] × 100.

### 2.10. Apoptosis Assay

Apoptosis was detected using the Annexin V-FITC/PI Apoptosis Detection Kit (Vazyme, code no. A211-01). Cells were harvested, washed with cold PBS, resuspended in 100 μL 1× Binding Buffer, and stained with 5 μL Annexin V-FITC and 5 μL PI. After incubating at room temperature for 10 min away from light, 400 μL Binding Buffer was added, and samples were analyzed by flow cytometry (FACS, Aria, BD) within 1 h. Flow cytometry data were analyzed using Flowjo software (V10, FlowJo, Ashland, OR, USA).

### 2.11. Cell Cycle Analysis

Cell cycle distribution was assessed using the Cell Cycle and Apoptosis Analysis Kit (Beyotime, code no. C1052, Shanghai, China). Cells were fixed in 70% ethanol (4 °C, 12-24 h), washed, and stained with 500 μL PI/RNase A solution (37 °C, 30 min). The flow cytometer detected red fluorescence at the excitation wavelength of 488 nm and simultaneously measured light scattering. Appropriate analysis software was used for the analysis of cell DNA content and light scattering.

### 2.12. Bioinformatics Analysis

Potential miRNAs and target genes of circRNA_4083 were predicted using Miranda (http://cbio.mskcc.org/microrna_data/miRandaaug2010.tar.gz) (accessed on 15 January 2023) (https://github.com/hacktrackgnulinux/miranda?tab=readme-ov-file) (accessed on 15 March 2023) and DIANA Tools (https://dianalab.e-ce.uth.gr/html/dianauniverse/index.php?r=microT_CDS) (accessed on 15 January 2025). A circRNA_4083/miRNA/mRNA competing endogenous RNA (ceRNA) network was visualized with Cytoscape 3.7.1 (https://cytoscape.org/) (accessed on 15 January 2025). Functional enrichment analysis (GO and KEGG pathways) was performed using DAVID (https://david.ncifcrf.gov/) (accessed on 18 January 2025).

### 2.13. Statistical Analysis

Data are presented as mean ± SEM. Statistical significance was analyzed using one-way ANOVA (for comparisons involving > 2 groups) or the independent samples *t*-test (for two-group comparisons) with SPSS 26.0 (SPSS Inc., Chicago, IL, USA) and GraphPad Prism 10 (GraphPad Software Inc., San Diego, CA, USA); non-normally distributed data were analyzed using the Mann–Whitney U test. All experiments were performed in triplicate (* *p* < 0.05, ** *p* < 0.01, *** *p* < 0.001, **** *p* < 0.0001).

## 3. Results

### 3.1. Characterization of Chicken-Derived circRNA_4083

**Figure 1:**
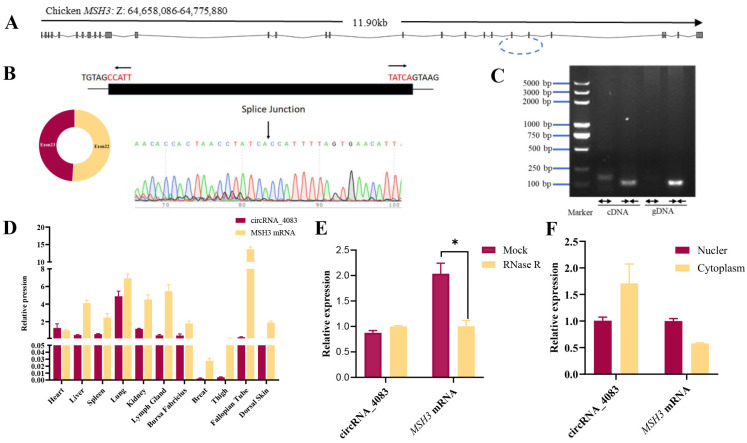
Characterization of chicken-derived circRNA_4083. (**A**) Genomic locus of circRNA_4083 within the *MSH3* gene, comprising exons 22 and 23 (225 bp in total). (**B**) Sanger sequencing validation of the circRNA_4083 splice junction. (**C**) Divergent primers (outward arrows) amplified circRNA_4083 from cDNA but not genomic DNA (gDNA), while convergent primers (inward arrows) amplified linear isoforms from both cDNA and gDNA. (**D**) Tissue-specific expression profiles of circRNA_4083 and *MSH3* mRNA in normal chicken tissues by qRT-PCR. (**E**) qRT-PCR analysis of circRNA_4083 and *MSH3* mRNA abundance following RNase R exonuclease treatment. (**F**) Subcellular localization of circRNA_4083 and *MSH3* mRNA by qRT-PCR, normalized to nuclear expression levels. Data are expressed as mean ± SEM (n = 3). Group differences were analyzed using the Student’s *t*-test (two-group comparisons) or one-way ANOVA (multi-group comparisons). (* *p* < 0.05).

Based on the previously reported transcriptome dataset GSE118752 [10], it was identified that circRNA_4083 exhibited differential expression. Consequently, further validation and in-depth analysis were undertaken. Genomic structure analysis revealed that circRNA_4083 was an exon-derived circular RNA (circRNA) composed of exons 22 and 23 of the *MSH3* gene, totaling 225 base pairs (Figure 1A). The PCR product was amplified and confirmed through Sanger sequencing (Figure 1B), with the arrow above the peak indicating the splicing junction of circRNA_4083. As illustrated in Figure 1C, divergent primers specifically generated the circRNA isoform of circRNA_4083 from cDNA but not from gDNA, whereas convergent primers could amplify both the linear isoform of circRNA_4083 from both cDNA and gDNA. Subsequently, we investigated the expression pattern of circRNA_4083 across various chicken tissues and cells. The tissue expression analysis showed that circRNA_4083 and *MSH3* mRNA were expressed across multiple tissues. circRNA_4083 displayed relatively high expression in the heart, lungs, and kidneys, whereas *MSH3* mRNA was expressed at higher levels than circRNA_4083 in most tissues (Figure 1D).

RNase R resistance assay further confirmed the circular stability of circRNA_4083. qRT-PCR analysis revealed that circRNA_4083 was highly resistant to RNase R treatment, whereas linear *MSH3* transcripts were significantly degraded (Figure 1E). Subcellular fractionation analysis by qRT-PCR showed that *MSH3* mRNA and circRNA_4083 were both distributed in the nucleus and cytoplasm, but circRNA_4083 was more abundant in the cytoplasm (Figure 1F). This specific distribution of circRNA_4083 may indicate its potential role as a competitive endogenous RNA (ceRNA). Collectively, these findings established circRNA_4083 as a stable and abundantly expressed circRNA in chicken tissues and cells.

### 3.2. Circularization Mechanism of circRNA_4083

**Figure 2:**
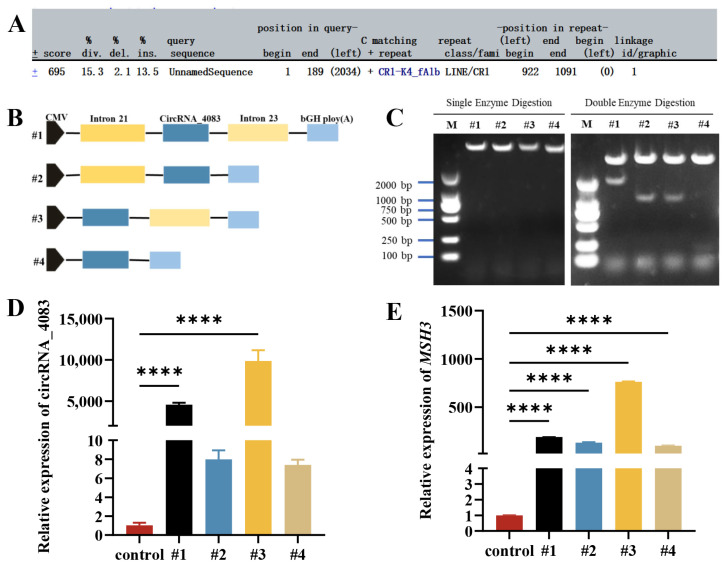
Circularization mechanism of circRNA_4083. (**A**) RepeatMasker prediction of repetitive elements in the flanking intronic sequences of circRNA_4083. (**B**) Schematic design of circRNA_4083 overexpression plasmids (#1–#4) containing varying genomic sequences. Plasmid #1 includes the genomic region of circRNA_4083 with upstream and downstream intronic sequences cloned into the pcDNA3.1 vector, while plasmids #2–#4 are deletion mutants constructed based on the *MSH3* expression plasmid. (**C**) Restriction enzyme digestion verification of the four successfully constructed plasmids. (**D**) qRT-PCR analysis of circRNA_4083 expression in DF-1 cells transfected with plasmids #1–#4. (**E**) qRT-PCR analysis of *MSH3* expression in transfected DF-1 cells. Data are expressed as mean ± SEM (n = 3). Group differences were analyzed using the one-way ANOVA (multi-group comparisons). (**** *p* < 0.0001).

Next, we focused on exploring the circularization mechanism of circRNA_4083 and constructing its overexpression vector. First, we used the RepeatMasker Web Server (http://www.repeatmasker.org/) (accessed on 20 January 2025) to predict repetitive sequence features in the flanking intronic regions of circRNA_4083. The results indicated the presence of only simple repeat sequences in the flanking regions (Figure 2A). Subsequently, we employed molecular cloning techniques to construct deletion plasmids as illustrated in Figure 2B. We successfully obtained the full-length sequence of circRNA_4083 (225 bp). Next, we amplified approximately 1000 bp upstream and downstream sequences via PCR and ligated them to circRNA_4083. Finally, four plasmids (#1, #2, #3, and #4) were successfully constructed by inserting these fragments into the pcDNA3.1(+) vector (Figure 2C). These plasmids were then transfected into DF-1 cells to assess gene expression changes. Quantitative PCR analysis revealed that circRNA_4083 overexpression of all plasmids upregulated both circRNA_4083 and *MSH3* gene expression when compared with the control group. Notably, plasmid #3 (containing circRNA_4083 and intron 23) exhibited the highest expression levels (Figure 2D,E). These findings demonstrate that the circularization of circRNA_4083 depends on reverse complementary repeat sequences in its flanking regions.

### 3.3. circRNA_4083 Suppresses Apoptosis and Promotes Cell Proliferation

**Figure 3:**
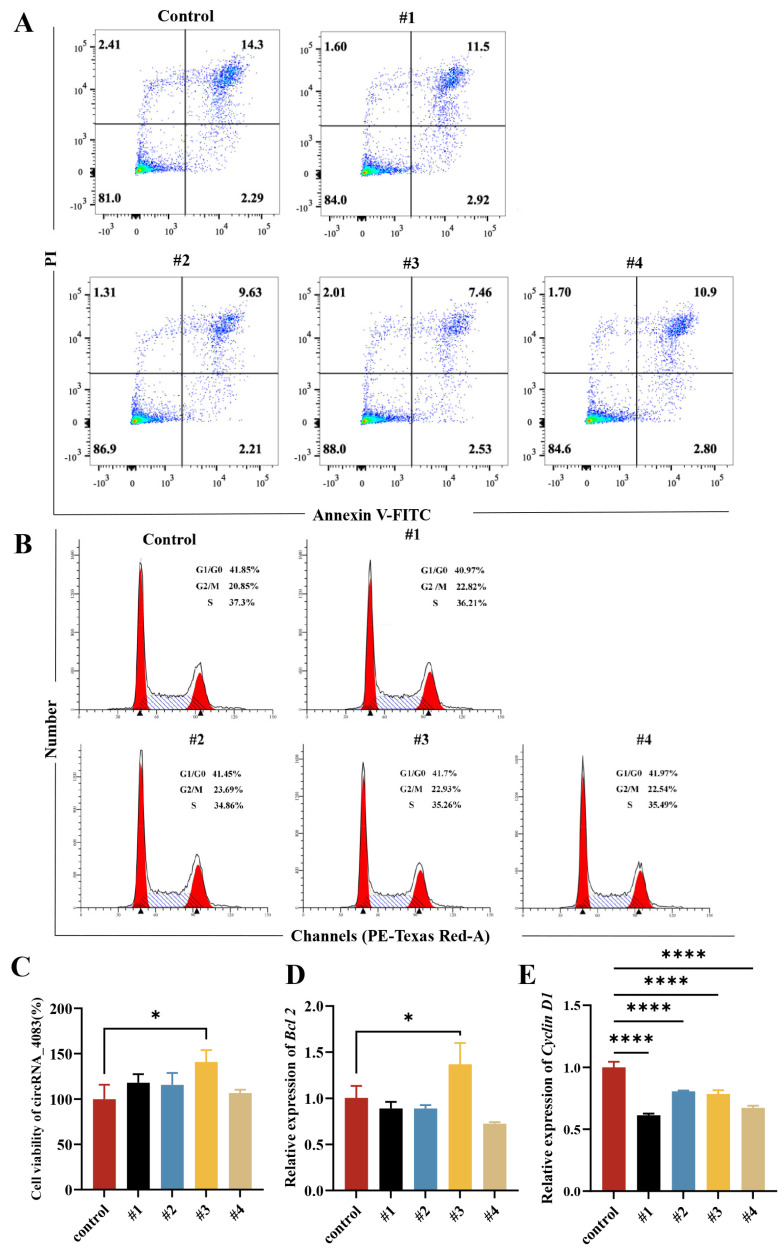
Overexpression of CircRNA_4083 down-regulates the level of cell apoptosis and promotes cell proliferation. The control was transfected with the pcDNA3.1 empty vector, #1 was transfected with the plasmid containing the genomic region of circRNA_4083 and its upstream and downstream intronic sequences cloned into the pcDNA3.1 vector, and #2 to #4 were transfected with deletion mutants constructed based on the *MSH3* expression plasmid. (**A**) Flow cytometry analysis of apoptosis in DF-1 cells. (**B**) Flow cytometry-based cell cycle profiling. (**C**) Statistical analysis of cell viability. (**D**) qRT-PCR detection of apoptosis-related gene *Bcl2*. (**E**) qRT-PCR detection of cell cycle-related gene *Cyclin D1*. Data are expressed as mean ± SEM (n = 3). Group differences were analyzed using the one-way ANOVA (multi-group comparisons). (* *p* < 0.05, **** *p* < 0.0001).

After successfully constructing four circRNA_4083 overexpression plasmids #1, #2, #3, and #4, these plasmids were transfected into DF-1 cells to examine changes in cell viability and apoptosis. Apoptosis detection results showed that, when compared with the control group, circRNA_4083 overexpression significantly inhibited late apoptosis in DF-1 cells, with plasmid #3 exhibiting the most pronounced inhibitory effect (Figure 3A). To determine cell cycle alterations after circRNA_4083 overexpression, flow cytometry analysis was performed. In comparison with the control group, the overexpression of circRNA_4083 elevated the proportion of cells in the G2/M phase, suggesting that cell proliferation was promoted to a certain degree. (Figure 3B). Cell viability assays revealed that circRNA_4083 overexpression upregulated DF-1 cell viability, promoting cell proliferation, with plasmid #3 showing the most significant enhancement (Figure 3C). To further validate the function of circRNA_4083, quantitative real-time PCR was conducted to measure the expression levels of apoptosis-related gene (*Bcl2*) and cell cycle-related genes (*Cyclin D1*) in transfected DF-1 cells. Overexpression of circRNA_4083 led to a significant increase in the expression level of the *Bcl2* gene (Figure 3D). Overexpression of circRNA_4083 led to a significant decrease in the expression of *Cyclin D1* gene, which was consistent with the previously observed increase in the proportion of cells in G2/M phase (Figure 3E). These results suggest that overexpression of circRNA_4083 can down-regulate the apoptotic level of DF-1 cells and promote cell proliferation.

**Figure 4:**
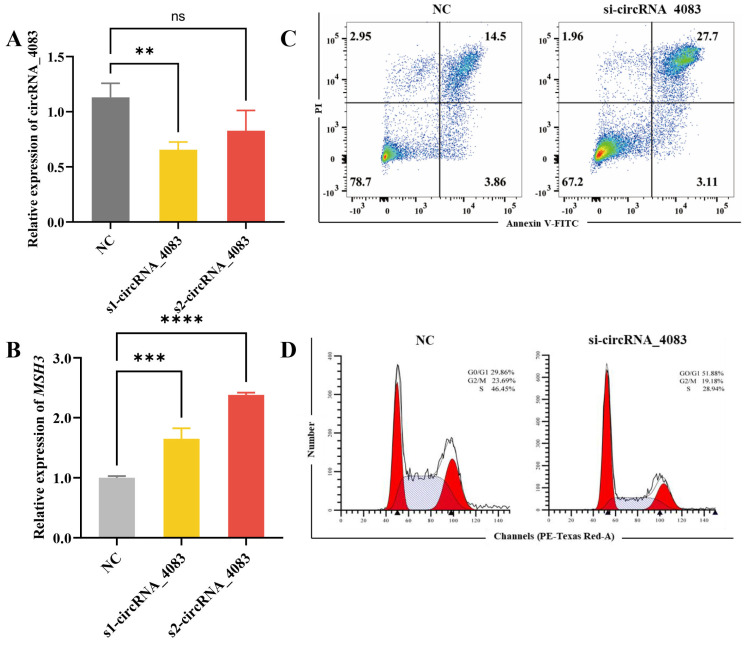
The silencing of CircRNA_4083 can upregulate the level of cell apoptosis and inhibit cell proliferation. (**A**) The interference efficiency after transfection of different siRNA-circRNA_4083 in DF-1 cells. (**B**) qRT-PCR analysis of *MSH3* expression after transfection of different siRNA-circRNA_4083 in DF-1 cells. (**C**) Apoptosis of DF-1 cells after transfection with siRNA-circRNA_4083. (**D**) The cell cycle of DF-1 cells after transfection with siRNA-circRNA_4083. Data are expressed as mean ± SEM (n = 3). Group differences were analyzed using the one-way ANOVA (multi-group comparisons). (ns, *p* > 0.05, ** *p* < 0.01, *** *p* < 0.001, **** *p* < 0.0001).

To further investigate the function of circRNA_4083, we transfected two distinct siRNAs targeting circRNA_4083 into DF-1 cells to suppress its expression. The expression level of circRNA_4083 was detected by qRT-PCR. The results show that s1-circRNA_4083 (circRNA_4083 siRNA1) exhibited superior interference efficiency and significantly reduced the expression level of circRNA_4083 (Figure 4A). The knockdown of circRNA_4083 did not alter the abundance of linear *MSH3* mRNA (Figure 4B), which confirms that the siRNA specifically targeted the circular isoform without affecting its source gene. Consequently, s1-circRNA_4083 was selected for subsequent experiments. Flow cytometry analysis of apoptosis demonstrated that si-circRNA_4083 significantly enhanced late apoptosis (Figure 4C). Furthermore, cell cycle analysis indicated that si-circRNA_4083 markedly decreased the proportions of cells in the S and G2/M phases while significantly increasing the percentage of cells in the G1/G0 phase (Figure 4D). These results indicate that interfering with the expression of circRNA_4083 can increase the apoptosis level of DF-1 cells and inhibit cell proliferation. This is contrary to the result of the overexpression of circRNA_4083.

In summary, circRNA_4083 inhibits apoptotic activity and promotes cell proliferation in DF-1 cells.

### 3.4. circRNA_4083 May Indirectly Regulate DNA Repair and Genomic Stability

**Figure 5:**
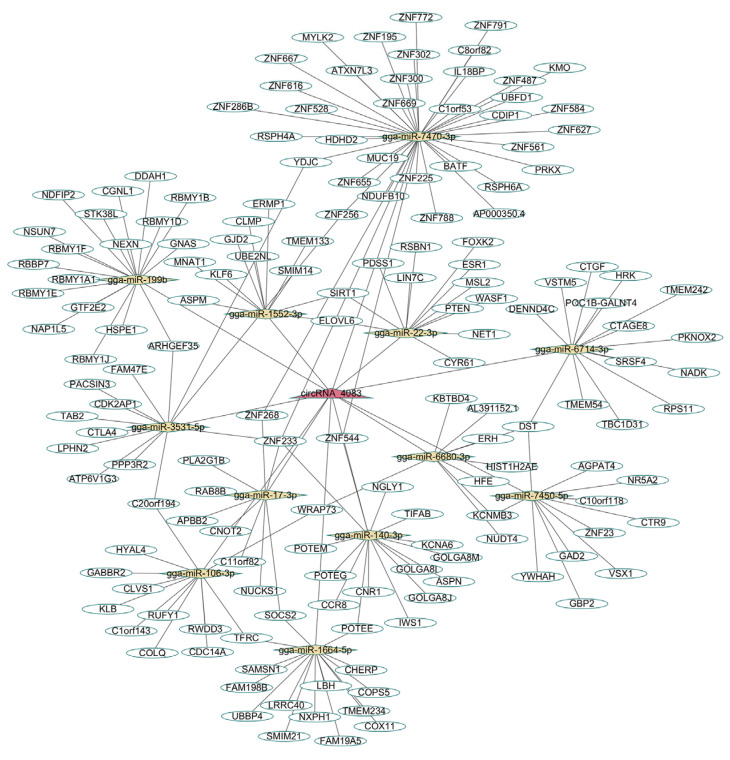
ceRNA (circRNA_4083-miRNA-mRNA) network diagram.

To further explore the potential functions of circRNA_4083, Miranda was used to predict its targeting miRNAs and mRNAs. A total of 12 miRNAs were predicted, and these miRNAs were subsequently employed to identify potential target genes. Using miRDB, TargetScan, and DIANA Tools, 2132 genes were predicted as targets for the aforementioned 12 miRNAs. After filtering these genes by retaining those with a miTGscore > 0.9, 209 genes were selected. A circRNA_4083-miRNA-mRNA regulatory network was constructed using Cytoscape (Figure 5), suggesting that circRNA_4083 may exert broader regulatory roles.

**Figure 6:**
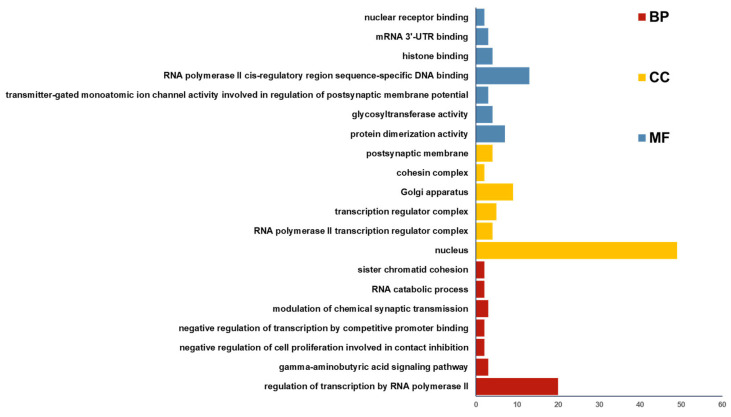
GO enrichment analysis of the predicted genes of 12 miRNAs.

**Figure 7:**
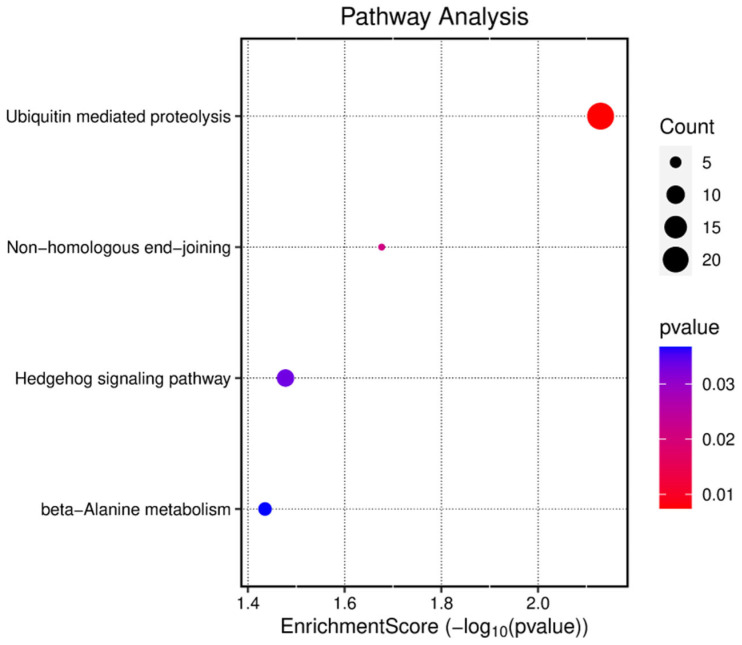
KEGG enrichment analysis of the predicted genes of 12 miRNAs.

Functional annotation of the predicted 2132 genes was performed through gene ontology (GO) and Kyoto Encyclopedia of Genes and Genomes (KEGG) enrichment analyses. GO analysis categorized gene functions into three aspects: molecular function (MF), cellular component (CC), and biological process (BP). As shown in Figure 6, the target genes of circRNA_4083 were primarily enriched in RNA polymerase transcriptional regulation, cell nucleus, Golgi apparatus, and mRNA binding. KEGG analysis revealed that the target genes were mainly enriched in pathways such as ubiquitin-mediated proteolysis, muscle cell cytoskeleton regulation, Hedgehog signaling pathway, and non-homologous end joining (Figure 7). Bioinformatics analysis revealed that circRNA_4083-associated genes were enriched in DNA repair pathways (e.g., non-homologous end joining), suggesting its potential indirect role in maintaining genomic stability, possibly through modulating *MSH3*-related mismatch repair processes.

## 4. Discussion

With the advancement of high-throughput sequencing technologies, circRNAs have emerged as a focal point in the field of non-coding RNA research. CircRNAs exhibit high stability and regulate cellular physiological activities through diverse mechanisms. circRNA_4083 is abundantly expressed in most chicken tissues and demonstrates stable and efficient expression. The relatively low levels of its associated linear mRNA may result from splicing competition between the circRNA and pre-mRNA. Zhang et al. have reported that circRNAs modulate gene expression by competing with linear splicing and identified muscleblind-like protein (MBL) as a key factor in circRNA biogenesis [21] Flanking intronic regions of circRNAs in Caenorhabditis elegans and humans are enriched with reverse complementary motifs (RCMs), which facilitate circularization [22]. Sequence analysis of circRNA_4083 revealed that it consistency with the previously observed circularization depended on reverse complementary repeats. This observation may reflect species-specific differences in genomic repeat content: mammalian genomes contain 40–50% repetitive sequences, whereas avian genomes harbor only 10%.

Extensive studies have revealed a close association between circular RNAs (circRNAs) and cellular viability as well as apoptosis. Zeng et al. have demonstrated that circHIPK2 was persistently upregulated in colorectal cancer (CRC) and inflammatory bowel disease (IBD). Silencing circHIPK2 significantly suppressed CRC cell growth in both in vitro and in vivo models [23]. Wang et al. reported that circRNA hsa_circ_0000848 promotes trophoblast cell migration and invasion while inhibiting apoptosis by sponging hsa-miR-6768-5p [24]. In hepatocellular carcinoma (HCC), Yang et al. identified circPTPN12 as a suppressor of cell proliferation. Mechanistically, circPTPN12 interacts with the PDZ domain of PDLIM2 to facilitate P65 ubiquitination, thereby inhibiting NF-KB signaling pathway activation [25]. To further explore the role of circRNA_4083, its effects on cell viability and apoptosis were evaluated in DF-1 cells. Functional studies of circRNA_4083 revealed that its silencing significantly increased cellular apoptosis while reducing S/G2-M phase cell populations. Conversely, overexpression decreased apoptotic levels and elevated G2-M phase cell proportions. These findings indicate that CircRNA_4083 suppresses apoptosis and promotes cell proliferation. Similarly, circITCH acts as a sponge for miR-7 and miR-214, inhibiting gastric cancer cell apoptosis through modulation of downstream signaling pathways [26]. CircACC1 could directly bind to the β and γ subunits of AMP-activated protein kinase (AMPK), thereby enhancing the stability and enzymatic activity of the AMPK holoenzyme. This interaction promotes cellular glycolytic capacity and fatty acid oxidation, which enables cells to sustain normal metabolic functions and maintain cellular viability under energy stress conditions [27]. Notably, circXRN2 is highly expressed in colorectal cancer and functions as a sponge for miR-149-5p, alleviating miR-149-5p-mediated repression of its target genes. This mechanism inhibits apoptosis and promotes CRC cell proliferation and invasion [28]. These findings, consistent with the results of the present study, collectively demonstrate that circRNAs broadly enhance cellular viability and suppress apoptotic processes.

A substantial body of research has demonstrated that circular RNAs (circRNAs) play pivotal roles in cellular growth, cancer, tumorigenesis, and other pathophysiological processes. For instance, circMEF2A positively regulates the differentiation of chicken skeletal muscle satellite cells in vitro and skeletal muscle development in vivo via the competing endogenous RNA (ceRNA) mechanism [29]. Hsa_circ_0005185 interacts with OTUB1 and RAB8A, facilitating the binding of the deubiquitinating enzyme OTUB1 to RAB8A. This interaction promotes RAB8A deubiquitination, thereby stabilizing RAB8A. The stabilized RAB8A enhances primary cilia regeneration and increases the production of GLI3R, a known suppressor of Hedgehog signaling. Consequently, this cascade ultimately inhibits androgen receptor (AR) activity and mitigates the progression of castration-resistant prostate cancer [30]. CircACVR2A, which is highly expressed in hepatocellular carcinoma (HCC), functions as a miRNA sponge by interacting with miR-511-5p to modulate the expression of proteins in the PI3K-Akt signaling pathway, thereby driving HCC cell proliferation, migration, and invasion [31]. Through bioinformatics analysis, we predicted 12 miRNAs and computationally identified 2132 potential target genes. Gene ontology (GO) and Kyoto Encyclopedia of Genes and Genomes (KEGG) pathway analyses revealed significant enrichment in pathways associated with cell growth and proliferation, including RNA polymerase transcription regulation, Hedgehog signaling, and non-homologous end joining (NHEJ). The Hedgehog signaling pathway is a canonical regulator of embryonic development and critically governs post-embryonic cell growth and proliferation. Dysregulation of Hedgehog signaling (either suppression or hyperactivation) is implicated in carcinogenesis. For example, Riobo-Del Galdo et al. demonstrated that circRNAs might regulate the Hedgehog pathway through multiple mechanisms, influencing breast cancer initiation and progression [32]. Gu et al. reported that circIPO11 activates Hedgehog signaling to drive self-renewal of liver cancer stem cells (CSCs), thereby promoting HCC proliferation and metastasis [33]. Similarly, Chen et al. found that Circ-GLI1 interacts with p70S6K2 to activate both Hedgehog/GLI1 and Wnt/β-catenin signaling pathways, upregulating CYR61 expression and enhancing melanoma metastasis and invasiveness [34]. Integration of these findings suggests that circRNA_4083 may indirectly participate in DNA repair and the maintenance of genomic stability.

## 5. Conclusions

circRNA_4083 is a highly abundant and stable circular RNA transcript. Its circularization is dependent on the flanking of reverse complementary sequences. Functional studies demonstrate that circRNA_4083 promotes cell proliferation and inhibits apoptosis. Bioinformatics analyses further suggest its potential indirect involvement in DNA repair and the maintenance of genomic stability.

## Figures and Tables

**Table 1:** qRT-PCR primer sequence.

Gene	Primer Sequence (5′-3′)
CircRNA_4083	F:CTACTGGGAGAACAGGATCA
	R:TTATCTTTCACAACTGGTCTGC
*MSH3*	F:CTGTATCTAAAGCGATTGGTC
	R:GCTGATTATCTTTCACAACTGG
*GAPDH*	F:GAACATCATCCCAGCGTCCA
	R:CGGCAGGTCAGGTCAACAAC

**Table 2:** Sequence of primer for vector construction.

Plasmid	Primer Sequence (5′-3′)
#1-intron21	F:agcgtttaaacttaagcttggtaccGCACTGGAATAGGCTCCCCG
	R:actaaaatggctctacaccaAAACAGGGAAAATAGAAAGAGG
#1-4083	F:tggtgtagagccattttagtGAACATTATCACACTGTATCTAAAGC
	R:caatacttactgtgataggtTAGTGGTGTTTGGAACATACT
#1-intron23	F:accatgaatgacactatttgAGATACCCTTTGAGCTTCAAATCA
	R:aacgggccctctagactcgaGTCTCCTGCTGCAACACCAATATCTG
#2-4083	F:tggtgtagagccattttagtGAACATTATCACACTGTATCTAAAGC
	R:aacgggccctctagactcgagtgTGATAGGTTAGTGGTGTTTGGAACATACTG
#3-4083	F:agcgtttaaacttaagcttgGTACCCCATTTTAGTGAACATTATCACACTGTATCTAAAGC
	R:caatacttactgtgataggtTAGTGGTGTTTGGAACATACT
#4	F:agcgtttaaacttaagcttggtaccCCATTTTAGTGAACATTATCACACTGTATCTAAAGC
	R:aacgggccctctagactcgagtgTGATAGGTTAGTGGTGTTTGGAACATACTG

Note: Lowercase letters are overlapping sequences.

**Table 3:** Target sequence of siRNAs.

Name of siRNA	Primer Sequence (5′-3′)
s1-circRNA_4083	CACTAACCTATCACCATTT
s2-circRNA_4083	AACCTATCACCATTTTAGT

## Data Availability

All datasets generated or analyzed during this study are available from the corresponding author on reasonable request.

## Abbreviations

The following abbreviations are used in this manuscript:

ALV-J	Avian leukosis virus subgroup J
*MSH3*	MutS Homolog 3
DF-1	Douglas Foster-1
